# Determinants of the serial changes in measurements of renal allograft Doppler resistive index in the first postoperative month

**DOI:** 10.1590/2175-8239-JBN-2018-0232

**Published:** 2020-05-18

**Authors:** José A. Moura-Neto, Ana Flávia Moura, José Hermógenes Rocco Suassuna, Nordeval Cavalcante Araújo

**Affiliations:** 1 Universidade do Estado do Rio de Janeiro, Divisão de Nefrologia, Rio de Janeiro, RJ, Brasil.; 2 Grupo CSB, Salvador, BA, Brasil.

**Keywords:** Ultrasonography, Doppler, Kidney Transplantation, Allografts, Ultrassonografia Doppler, Transplante de Rim, Aloenxertos

## Abstract

**Introduction:**

The role of single Doppler-derived renal resistive index (RI) in renal allograft management is still a controversial issue, however detection of changes in serial duplex scanning has been reported as more valuable. This study aimed to test the hypothesis that early change in RI following transplantation may be related to factors associated with delayed graft function (DGF).

**Material and methods:**

113 patients were included, in whom two RI measurements were performed within 30 days post-transplant. According to an RI change (equal to or more than 10%) in the second measurement, patients were assigned to decrease (Group I), no change (Group II), or increase (Group III) group.

**Results:**

30 subjects had a decrease, 55 had no change, and 28 had an increase in the second RI measurement. The donors were younger in Group III in comparison to Group II. In comparison to Group I, Group III had a higher frequency of deceased donor, DGF, and presence of tubular necrosis and tubular vacuolization in peri-implantation biopsies.

**Conclusion:**

the increase of RI during the first weeks of the postoperative period seems to be associated with DGF and with tubular necrosis / tubular vacuolization in peri-implantation biopsies, likely related to ischemia reperfusion injury.

## Resumo

**Introdução:**

O papel do índice de resistividade renal (IR) derivado de varredura por Doppler no manejo de aloenxertos renais, em exame isolado, ainda é uma questão controversa; no entanto, em exames seriados, a detecção de alterações nas imagens duplex tem sido relatada como mais relevante.

**Material e métodos:**

113 pacientes foram incluídos, nos quais duas medidas de IR foram realizadas dentro de 30 dias após o transplante. De acordo com uma alteração do IR (igual ou superior a 10%) na segunda medida, os pacientes foram classificados em redução (Grupo I), nenhuma alteração (Grupo II) ou aumento (Grupo III).

**Resultados:**

30 indivíduos tiveram redução, 55 não tiveram alterações e 28 tiveram aumento na segunda medição do IR. Os doadores eram mais jovens no Grupo III em comparação ao Grupo II. Em comparação ao Grupo I, o Grupo III apresentou maior frequência de doador falecido, FTE, presença de necrose tubular e vacuolização tubular nas biópsias peri-implantares.

**Conclusão:**

o aumento do IR durante as primeiras semanas no período pós-operatório parece estar associado à FTE e à necrose tubular/vacuolização tubular nas biópsias peri-implantares, provavelmente relacionadas à lesão por isquemia-reperfusão.

## Introduction

Renal transplantation is still recognized as the best choice for end stage renal disease treatment^1^. However, renal allograft recipients have a high risk of surgical complications, mainly vascular, in the immediate post-operative period that requires careful surveillance to detect them as early as possible. Doppler sonography is by far the most commonly used imaging method to assess anatomical allograft integrity^2^^,^ 3. Moreover, the Doppler-derived renal resistive index (RI) has been proposed to estimate renal blood flow^3^.

As many factors influence RI value, the role of a single RI measurement for assessment of renal allograft status remains uncertain^4^. On the other hand, detection of changes in serial duplex scanning has been reported as more valuable for diagnosing allograft dysfunction^5^. However, this issue is still a matter of debate^6^^-^ 8, rendering the interpretation of RI difficult in transplantation practice.

A comprehensive understanding of determinant factors of RI in early transplant has been reported as valuable in the interpretation of the RI value^9^. In order to shed light on this issue, the current study aimed to test the hypothesis that the change in RI within the first weeks following transplantation may be related to factors associated with delayed graft function (DGF).

## Material and methods

The institutional review committee approved the study protocol and consent was waived due to the retrospective nature of the study. The study design was retrospective, based on two samples. All patients with end-stage renal disease who underwent a living-donor or deceased-donor renal transplant during this time were recruited. One hundred patients with renal allografts from deceased donors and thirteen from living donors were included, in whom two investigations of the kidney allograft using color Doppler ultrasound were performed within thirty days post-transplant, independently of the occurrence of delayed graft function.

The exclusion criteria were: hydronephrosis of grade 2 or higher; low-quality renal sonography (less than two interlobar arteries insonation); peri-renal fluid collections with marked compression; and histological diagnosis of acute rejection. In case of retransplant, only the second renal transplant episode during the study period was included; results, therefore, reflect one transplant procedure per study patient.

All ultrasounds were performed by the same operator (NCA) using a Sonoline 40 (Erlangen, Germany) instrument with a 3.5 MHz transducer. A complete description of sampling has been already explained elsewhere^9^. Briefly, we measured the longitudinal diameter, and RI was sampled at the level of the interlobar artery. The RI was manually measured with built-in software. An average of at least two, and in most cases three, samplings was obtained. The mean RI value measured postoperatively in the first week and in the second, third, or fourth week was used for analysis.

Patients were assigned to decrease (Group I), no change (Group II), or increase (Group III) group according to an RI change in the second measurement in relation to the first. Accordingly, patients with a decrease equal to or more than 10% comprised Group I and an increase equal to or more than 10% comprised Group III. Patients with intermediate change in the second RI comprised Group II. Data from the three groups were compared for statistical differences. To ensure that the groups were similar, we included related variables such as those related to the graft. We compared the vintage dialysis (time span in months), recipient and donor sex, age and serum creatinine and pre-transplant panel-reactive antibodies (PRA), number of human leukocyte antigen (HLA) mismatches, *causa mortis*, and cold ischemia time (CIT). The number of HLA mismatches was calculated by adding the number of mismatches in the A, B, and DR loci. All patients received triple immunosuppression therapy, consisting of cyclosporine or tacrolimus, mycophenolate mofetil or azathioprine, and steroids. We also studied peri-implantation wedge biopsies. Glomerular obsolescence was quantified as the percentage of sclerotic glomeruli in relation to the total number. Interstitial fibrosis and tubular atrophy, interstitial infiltration and edema, vascular lesions (arteriolar hyalinosis, arteriolosclerosis, and fibrosis endarteritis), and acute tubular necrosis (ATN) were reported as present or absent. *Causa mortis* was dichotomized into trauma and other causes. Because of its retrospective nature, not all variables were available for every patient in the study. The primary cause of the chronic primary kidney disease was infrequently available in the patient’s medical record and, therefore, not reported in this paper. Delayed graft function was defined as the need for dialysis in postoperative period.

Results are presented as mean ± standard deviation (SD) for continuous and as percentage for dichotomous variables. Groups were compared with the ANOVA test for continuous variables followed by Bonferroni’s and Chi-square tests for categorical variables. Significant differences between groups were indicated by a *p*-value less than 0.05.

## Results

One hundred and thirteen (69 men, 44 women) out of 116 consecutive cases were studied. Three cases were excluded: one because of RI first measurement was later than one week, another due to need for reoperation, and the final one following a clinical and histological diagnosis of acute rejection. Twelve patients underwent retransplantation. The mean age was 46.07 ± 13.79 years (range 14.0-74.0 years). The first RI measurement was performed in the 3.88 ± 1.56 (range 0-7) and the second in the 16.58 ± 5.28 (range 10-29) postoperative day. The interval between the first and second measurement was 12.71 ± 5.46 (range 5-26) days. The mean value of the RI was 0.74 ± 0.12 (range 0.42-1.00) in the first measurement and 0.74 ± 0.11 (range 0.49-1.00) in the second measurement.

Although cadaveric donor recipients, in comparison to living donor recipients, had a quite similar RI in the first measurement (0.74 ± 0.12 vs. 0.73 ± 0.10; *p* > 0.05) the RI was significantly higher in the second measurement (0.74 ± 0.11 vs. 0.67 ± 0.0 vs; *p* = 0.029).

**Figure 1:**
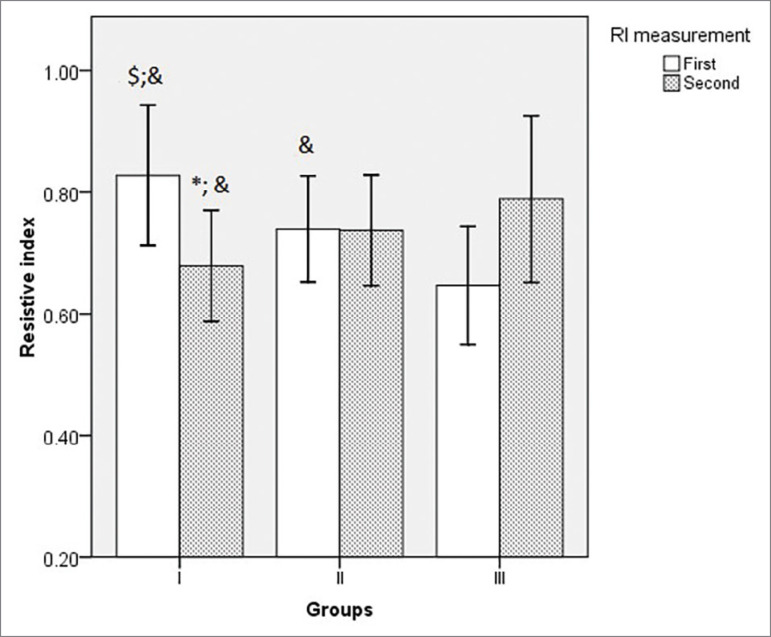
The mean and standard deviation value of RI according to the groups and time of measurement. ANOVA test: First measurement - ^$^ *p* < 0.005 vs. Group II; ^&^ *p* < 0.005 vs. Group III; Second measurement - *p < 0.05 vs Group II; ^&^ *p* < 0.005 vs. Group III.

According to the established criteria, 30 subjects had a decrease (Group I), 55 had no change (Group II), and 28 had an increase (Group III) in the second RI measurement in relation to the first. The mean value of the first RI was statistically higher in Group I than in Group II and Group III, and in Group II than in Group III, while the second RI measurement was lower in Group I than in Group II and Group III (Table 1). The donors were younger in Group III in comparison to Group II (Table 1). The mean and standard deviation values of RI according to the groups and time of measurements are depicted in graph to improve data comprehension (Figure 1).

**Table 1:** Continuous Variable: Clinical data, laboratory and conventional/Doppler ultrasound parameters in patients with decrease (Group I), no change (Group II) and increase (Group III) in the second RI (2) measurement in relation to the first (1)

	Group I (30)		Group II (55)		Group III (28)	
Variable	Mean	SD	Mean	SD	Mean	SD
Recipient age (y)	45.13	13.50	47.35	14.04	44.57	13.87
Dialysis vintage (mos)	70.17	56.55	75.69	60.11	76.20	43.06
Transplant time 1 (d)	4.07	1.14	3.98	1.71	3.25	1.71
Recipient Cr 1 (mg/dl)	7.02	2.11	6.72	2.87	6.69	2.38
Kidney length 1 (mm)	118.1	10.6	118.4	7.99	118.0	10.89
Intrarenal artery RI 1	0.832,4	0.12	0.744	0.09	0.64	0.09
Transplant time 2 (d)	17.97	5.13	15.73	5.28	16.79	5.27
Recipient Cr 2 (mg/dl)	3.693	2.30	4.13	2.49	5.58	2.31
Kidney length 2 (mm)	120.4	6.37	121.5	7.74	121.9	10.25
Intrarenal artery RI 2	0.681,4	0.09	0.74	0.09	0.79	0.14
PRA class I (%)	1.76	7.59	5.50	17.68	2.81	11.45
PRA class II (%)	4.97	17.29	6.58	18.77	3.44	17.50
Donor age (y)	39.55	15.55	45.30	12.18	34.752	14.74
Donor Cr (mg/dl)	1.45	0.64	1.25	0.62	1.37	0.95
CIT (min)	1296	308	1205	365	1393	386

ANOVA test, ^1^ *p* < 0.05 or ^2^ *p* < 0.005 vs. Group II; ^3^ *p* < 0.05 or ^4^ *p* < 0.005 vs. Group III; SD: standard deviation; Cr: plasma creatinine; RI: resistive index; PRA: panel reactive antibody; CIT: cold ischemia time; y: years; mos: months; d: days; min: minutes.

1: first measurement of the variable. 2: second measurement of the variable.

In comparison to Group I, Group III had a higher frequency of deceased donors, delayed graft function, and presence of composite parameter incorporating tubular necrosis and tubular vacuolization in peri-implantation biopsies (Table 2).

**Table 2:** Categorical variables: clinical data, HLA mismatches, and peri-implantation biopsy findings in patients with decrease (Group I), no change (Group II), and increase (Group III) in the second RI measurement in relation to the first

Variable	Group I (30)	Group II (55)	Group III (28)
	%	%	%
Recipient sex, male	70.0	60.0	53.6
Donor type, deceased	83.3	85.5	100.0*
Retransplant	6.70	5.50	14.30
Donor sex, male	60.0	38.9	53.6
Causa mortis, trauma	52.0	48.9	53.6
MM HLA, ≥ 3	53.3	37.7	28.6
Vascular abnormalities	37.5	31.6	18.2
Tubulointerstitial changes	50.0	72.7	30.8
Tubular necrosis (TN)	31.3	50.0	38.5
Tubular vacuolization (TV)	43.8	45.5	69.2
DGF	40.0	43.6	71.4*

Chi-square test, Groups I vs. III;

* *p* < 0.05; MM: mismatch; HLA: human leucocyte. DGF: delayed graft function.

There was no statistical difference among groups with respect to recipient age and serum creatinine, dialysis duration, time of the first and second RI measurements, kidney length in the first and second measurements, PRA class I and II, and cold ischemia time (Table 1). The differences among groups in terms of donor and receptor sex, retransplant, *causa mortis*, HLA mismatches, and histological lesions other than tubular necrosis/vacuolization found in biopsies were not statistically significant (*p* > 0.05) (Table 2). The percentage of patients who developed DGF stratified by group is also given in Table 2.

## Discussion

In the postoperative period, the surveillance of kidney allograft includes an approach to the diagnosis and management of delayed graft function due to ischemia reperfusion injury. The Doppler ultrasound is the imaging method of choice to closely monitor renal transplant^2^^,^ 3. Resistive index is a Doppler-derived parameter commonly used to evaluate blood flow integrity^10^. An abnormally high value for RI has been attributed to tubular acute necrosis and acute rejection^11^^,^ 12. However, the clinical utility for this tool is still based on conflicting reports^4^^,^ 13 ^,^ 14. Moreover, the role of a single measurement of RI in the evaluation of kidney allograft is controversial^5^^,^ 15. On the other hand, many studies suggest a high sensitivity and sensibility when a serial duplex index (SDI) is used in this analysis. Meier et al. observed that SDI was more accurate in identifying acute renal transplant rejection than RI and PI calculated at a single time-point^16^. In a retrospective study including 6017 serial duplex scans in 614 patients, SDI was better than one-time scanning in heralding the need for renal graft biopsy in the diagnosis of acute rejection^17^.

In this study the RI was measured on two occasions within the first four weeks following the surgical procedure. None of the patients performed endovascular procedure during the study period. The last RI measurement was performed at the 29^th^ postoperative day, a period in which transplant renal artery stenosis rarely develops^18^^-^ 20.

The main finding of the present study was that in a subset of patients who underwent deceased donor transplant, the RI increased in a subsequent measurement after the first week. This kind of change in RI was not found in patients who received a kidney from a living donor. In a univariate analysis, the additional determinant factors associated with this group of patients were the need for dialysis in the immediate postoperative period and the presence of tubular necrosis and/or tubular vacuolization in the perimplantation renal biopsy. The small number of events preclude a multivariate analysis to investigate the independent association between each one of these variables and the event of interest.

Taken together, these findings corroborate that the serial increase in RI is strongly associated with the development of a more severe type of ischemia reperfusion injury. In line with these results, several studies suggest a relationship between increased RI and delayed graft function, acute rejection, histologic lesions on biopsy, and transplant failure^7^^,^ 16 ^,^ 21 ^-^ 25. In agreement with the results of the current study, in which patients with decrease in RI (Group I) had better SCr results and lower likelihood to require dialysis in the observed period, other studies found a direct relationship between RI and serum creatinine^8^^,^ 26 ^,^ 27.

The younger age of donors in Group III in relation to Group II, although statistically not significant, probably reflects a higher percentage of *causa mortis* due to trauma (more common in younger people) in the former group.

The weakness of this study is that no biopsy was taken after transplantation (besides peri-implantation wedge biopsies) to better differentiate the groups. Therefore, it is possible that the early graft function and DGF subgroups were unequally contaminated by cases of acute rejection and/or acute tubular necrosis. However, it is well-known that using the current immunosuppressive regimen, the incidence of acute rejection in the first two weeks is as low as 2.28% (6/263)^28^ and the median time to acute rejection is 23 days^29^. The length of DGF was not a variable included in the study. In addition, some variables like hypertension and diabetes were not available for all donors and weight and height were not available at all. The missing variables render KDPI evaluation inaccurate.

In conclusion, the increase of RI during the first weeks of the postoperative period seems to be associated with DGF and with tubular necrosis / tubular vacuolization in peri-implantation biopsies, likely related to ischemia reperfusion injury.

A more comprehensive understanding of determinants of RI changes during the course of ischemia reperfusion following renal transplantation might decrease the need for percutaneous biopsy in some cases.

## Abbreviations

RI: resistive index.
DGF: delayed graft function.
HLA: human leukocyte antigen.
CIT: cold ischemia time.
ATN: acute tubular necrosis.
SD: standard deviation.
